# IL-6 Is Not Absolutely Essential for the Development of a TH17 Immune Response after an Aerosol Infection with *Mycobacterium tuberculosis* H37rv

**DOI:** 10.3390/cells10010009

**Published:** 2020-12-22

**Authors:** Kristina Ritter, Jan Christian Sodenkamp, Alexandra Hölscher, Jochen Behrends, Christoph Hölscher

**Affiliations:** 1 Infection Immunology, Research Centre Borstel, D-23845 Borstel, Germany; kritter@fz-borstel.de (K.R.); jansodenkamp@icloud.com (J.C.S.); ahoelscher@fz-borstel.de (A.H.); 2 Core Facility Fluorescence Cytometry, Research Centre Borstel, D-23845 Borstel, Germany; jbehrends@fz-borstel.de; 3 German Centre for Infection Research (DZIF), Partner Site Hamburg-Borstel-Lübeck-Riems, D-23845 Borstel, Germany

**Keywords:** tuberculosis, rodent, cytokines, IL-6, gp130, T cells

## Abstract

Anti-inflammatory treatment of chronic inflammatory diseases often increases susceptibility to infectious diseases such as tuberculosis (TB). Since numerous chronic inflammatory and autoimmune diseases are mediated by interleukin (IL)-6-induced T helper (TH) 17 cells, a TH17-directed anti-inflammatory therapy may be preferable to an IL-12-dependent TH1 inhibition in order to avoid reactivation of latent infections. To assess, however, the risk of inhibition of IL-6-dependent TH17-mediated inflammation, we examined the TH17 immune response and the course of experimental TB in IL-6- and T-cell-specific gp130-deficient mice. Our study revealed that the absence of IL-6 or gp130 on T cells has only a minor effect on the development of antigen-specific TH1 and TH17 cells. Importantly, these gene-deficient mice were as capable as wild type mice to control mycobacterial infection. Together, in contrast to its key function for TH17 development in other inflammatory diseases, IL-6 plays an inferior role for the generation of TH17 immune responses during experimental TB.

## 1. Introduction

Tuberculosis (TB) is one of the major causes of death worldwide [1]. In 2018, 10 million individuals newly developed active TB, and approximately 1.5 million people died of this infectious disease. Reduced numbers in CD4^+^ T cells in concomitant HIV infection [2,3] and the treatment of autoreactive inflammatory diseases with anti-inflammatory drugs [4,5] are linked to reactivation of latent TB. Therefore, cell-mediated immune responses are essential to control the growth of *Mycobacterium tuberculosis* (Mtb) in humans.

In experimental animal models, protective immune responses against Mtb infection also depend on CD4^+^ T cells within pulmonary granuloma [6,7]. Protection executed by these T helper type (TH1) cells is mediated by the type 1 cytokines interferon gamma (IFNγ) and tumor necrosis factor (TNF) [7,8,9] both of which capable to activate anti-mycobacterial effector mechanisms in macrophages [10]. In addition to a TH1 immune response, interleukin (IL)-17A-producing TH17 cells are additionally required for controlling primary infection with virulent Mtb [11]. Moreover, vaccine-induced TH17 cells patrolling the lung are capable to react immediately to a challenge with Mtb [12], and several vaccination strategies are aimed to induce protective long-term TH17 responses [13,14]. However, an unrestricted TH17 immune response during experimental TB may also lead to immunopathology [15].

In vitro, the differentiation of TH17 cells is instructed by a combination of cytokines amongst others IL-23 and IL-6 [16,17,18,19]. Because a TH17 immune response considerably determines many chronic and autoimmune inflammatory diseases [20,21,22], IL-23 and/or IL-6 are critically involved in the pathogenesis of these disorders [23,24]. Experimental enterocolitis in IL-10-deficient (^−/−^) mice, for example, which is characteristic for chronic inflammatory bowel disease in humans, is dependent on IL-23-induced IL-17A-producing cells [25]. In experimental autoimmune encephalomyelitis (EAE), which is a model for multiple sclerosis, the differentiation of autoreactive TH17 cells is induced by IL-6 and its pathogenicity was demonstrated in IL-6^−/−^ mice which are completely resistant to the induction of EAE [26,27,28]. After infection with Mtb, IL-23 is indispensable for developing a TH17 immune response [29,30]. A role for IL-6 for protective immune responses in experimental TB has been shown after a high dose intravenous infection with Mtb [31]. However, whether IL-6 also participates in the differentiation and expansion of TH17 cells after aerosol infection with Mtb has not been elucidated so far.

To clarify the effect of IL-6 on the development of a TH17 immune response, we analyzed in this study the T cell response during the course of Mtb infection in IL-6^−/−^ mice. IL-6 uses the gp130/IL‑6R receptor complex for signaling and has pleiotropic effects on other cells [32] than T cells. Therefore, we additionally included T cell-specific gp130-deficient (CD4^cre^; gp130^loxP/loxP^) mice in our study.

## 2. Materials and Methods

### 2.1. Mice

CD4^cre^ and gp130^loxP/loxP^ mice [33] were kindly provided by Werner Müller (HZI; Braunschweig, Germany). IL-6^−/−^ mice on a C57BL/6 background [34] were purchased from the Jackson Laboratories (Bar Harbor, Maine). Mice 8 to 16 weeks of age were used in infection experiments. Animals of approximately the same age and sex were selected for any given experiment. Experimental mice for Mtb infection were then housed in individually ventilated cages in the BSL3 facility of the Research Center Borstel. The animal experiments were carried out in accordance with the German Animal Welfare Act, checked by the animal ethics board of the State of Schleswig-Holstein and approved by the Ministry of Energy, Agriculture, the Environment, Nature and Digitalization, Kiel, Germany (approval number 104/10-08).

### 2.2. Bacteria and Infection

For infection experiments, the Mtb strain H37Rv was used and aerosol infection was carried out using an inhalation exposure system (Glas-Col, Terre-Haute, IN, USA) as described [35].

### 2.3. Colony Enumeration Assay

To determine the bacterial load in the lungs, spleen and liver, the animals were sacrificed at different time points of Mtb infection, and the organs were removed, weighed and homogenized in PBS and further processed [35].

### 2.4. Histopathology and Immunohistochemistry

Lungs were fixed in 4% buffered formalin, embedded in paraffin and sectioned on a microtome. To examine histopathological changes during infection, sections were stained with hematoxilin/eosin. For the immunohistochemical detection of NOS2, sections were stained with a polyclonal rabbit anti-mouse NOS2 antiserum (Biomol, Hamburg, Germany) and examined microscopically.

### 2.5. RT-PCR

Before and at various time points of Mtb infection, weighed pieces of lungs were homogenized in 5 mL of 4 M guanidinium isothiocyanate buffer. After acid phenol extraction, RNA was then transcribed into cDNA and quantitative PCR was carried out on a light cycler (Roche Diagnostics Corporation, Indianapolis, IN, USA) as described [35]. The following primer and probe sets were employed. *Cxcl9:* sense 5′-CTT TTC CTC TTG GGC ATC AT-3′, antisense 5′-GCA TCG TGC ATT CCT TAT CA-3′, probe 5′-CCT GGA GC-3′; *Cxcl10:* sense 5′-GCT GCC GTC ATT TTC TGC-3′, antisense 5′-TCT CAC TGG CCC GTC ATC-3′, probe 5′-GGC AGG AG-3′; *Cxcl11:* sense 5′-TCT GCA AAG AGA GAT CTC CAA A-3′, antisense 5′-CGC CCC TGT TTG AAC ATA AG-3′, probe 5′-AGG CAG AG-3′; *Hprt:* sense 5′-TCC TCC TCA GAC CGC TTT T-3′, antisense 5′-CCT GGT TCA TCA TCG CTA ATC-3′, probe 5′-AGT CCA G-3′; *Il6*: sense 5′-GTC ACC AAA CTG GAT ATA ATC AGG A-3′, antisense 5′-CCA GGT AGC TAT GGT ACT CCA GAA-3′, probe 5′-TTC CTC G-3′; *Il10*: sense 5′-CAG AGC CAC ATG CTC CTA GA-3′, antisense 5′-TGT CCA GCT GGT CCT TTG TT-3′, probe 5′-CTT CAG CC-3′; *Il12b*: sense 5′-ATC GTT TTG CTG GTG TCT CC-3′, antisense 5′-GGA GTC CAG TCC ACC TCT ACA-3′, probe 5′-AGC TGG AG-3′; *Il27:* sense 5′-CAT GGC ATC ACC TCT CTG AC-3′, antisense 5′-AAG GCC CGA AGT GTG GTA-3′, probe 5′-CTG CTT CC-3′; *Lrg47*: sense 5′-AAG GCC ACT AAC ATC GAA TCA-3′, antisense 5′-TGC CTT ATC TCA CTT AAT ACT CCT CA-3′, probe 5′-CTC CTC TG-3′; *Nos2*: sense 5′-CTT TGC CAC GGA CGA GAC-3′, antisense 5′-TCA TTG TAC TCT GAG GGC TGA C-3′, probe 5′-AGG CAG AG-3′.

### 2.6. Preparation of Single Cell Suspensions from Infected Lungs

For flow cytometric analyzes and antigen-specific restimulation in the ELISPOT assay, single cell suspensions were obtained from lungs at different time points of infection [35]. For this purpose, the animals were anesthetized, and the lungs were removed from blood by perfusion with warm PBS through the right ventricle. To obtain single cell suspensions, the organs were digested, and cells were counted in an automatic cell counter (ViCell^®^; Beckman Coulter, Krefeld, Germany) and diluted for further experiments as described [35].

### 2.7. Flow Cytometry

For the detection of intracellularly expressed IFNγ and IL-17A in activated CD4^+^ T cells in lungs of Mtb-infected mice, an intracellular cytokine staining kit (BD Biosciences Heidelberg, Germany) was used according to the manufacturer’s instructions as described [35]. Briefly, 2 × 10^6^ cells of single cell suspensions were incubated with medium or with plate-bound anti-CD3/CD28 mAbs at 37 °C for 4 hrs in the presence of brefeldin A-containing GolgiPlug^TM^ (BD Biosciences). Subsequently, cells were incubated with anti-FcγRIII/II mAb (clone 2.4G2), mouse and rat serum, washed and surface markers were stained with anti-CD44-FITC (clone IM7) and anti-CD4-APC (clone RM4-5) (BD Biosciences). After subsequent fixation and permeablization in Cytofix/Cytoperm™ (BD Biosciences), intracellular IFNγ and IL-17A were detected with PE-labelled mAbs (clone XMG1.2 and clone TC11 18 H10, respectively) (BD Biosciences).

For the intracellular staining of FoxP3, the mouse regulatory T cell staining kit (eBioscience) was employed, and cell surfaces were additionally examined by staining with anti-CD4-FITC and anti-CD25-APC (BD Bioscience).

Fluorescence intensity was acquired on a FACSCanto II (BD Biosciences) (Figure S1).

### 2.8. ESAT6_1-20_-Specific ELISPOT Assays

The frequency of antigen-specific CD4^+^ T cells in infected lungs was determined in an ELISPOT assay after enrichment of CD4 T cells as described [35]. Briefly, antigen-specific production of IFNγ and IL-17A was quantified using an ELISPOT assay kit according to the manufacturer’s instructions (BD Bioscience and R&D Systems, Wiesbaden-Nordenstadt, Germany, respectively). Serially diluted CD4 T cells were added to mitomycin-D (Sigma-Aldrich, Hamburg, Germany)-inactivated spleen cells from uninfected wild type mice as antigen-presenting cells and incubated with Mtb ESAT6_1–20_ (10 µg/mL; Research Center Borstel, Germany) and recombinant mouse IL-2 (10 U/mL; Peprotech, Hamburg, Germany). After 20 hrs at 37 °C, the cells were washed, incubated with secondary mAbs. After development, spots were counted in an ELISPOT reader (EliSpot 04 XL; AID, Straßberg, Germany) and the frequency of the ESAT6_1–20_-specific CD4^+^ T cells was calculated.

### 2.9. Statistical Analysis

The individual results obtained were all shown as the mean value with standard deviation. Statistical analysis was performed using the Prism 8 software (GraphPad Software, San Diego, CA, USA).

## 3. Results

### 3.1. Mtb Infection of IL-6^−/−^ Mice

#### 3.1.1. The Absence of IL-6 has a Minor Effect on the Antigen-Specific TH17 Immune Response after Mtb Infection

**Figure 1:**
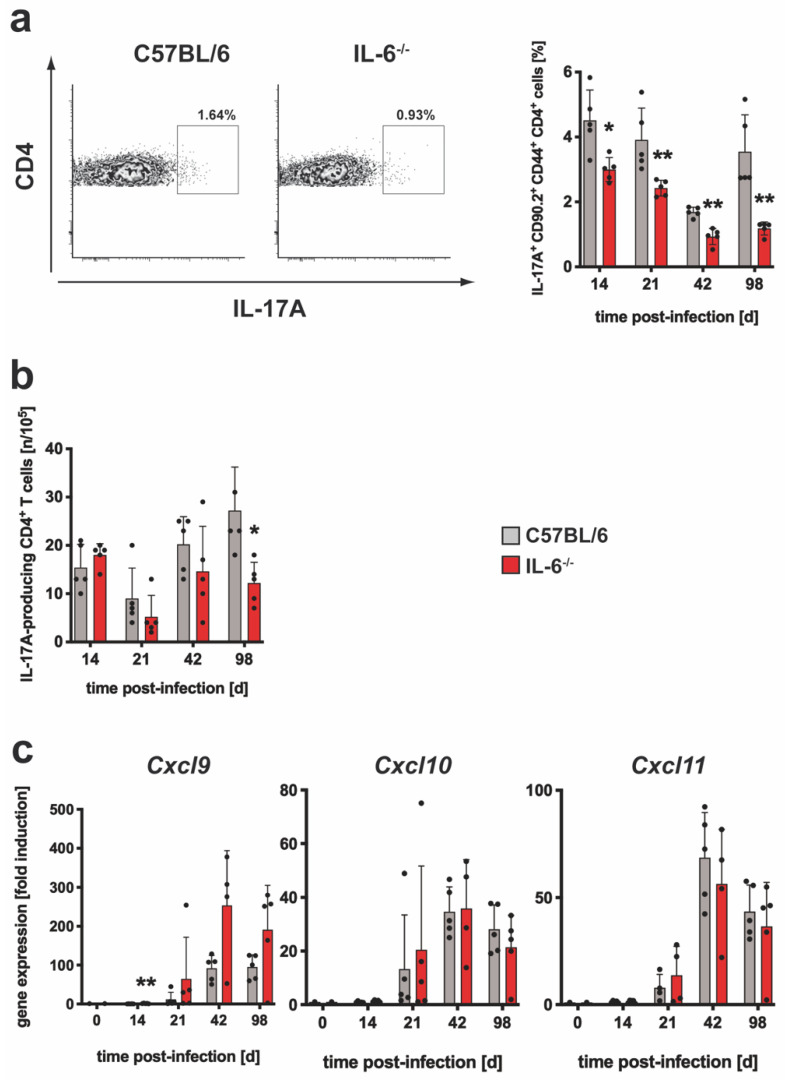
The TH17 immune response in IL-6^−/−^ mice during experimental TB. C57BL/6 (grey symbols) and IL-6^−/−^ (red symbols) mice were infected with approximately 100 CFU Mtb via the aerosol route. At the indicated time points, lungs were removed and further processed. (**a**) The relative amount of IL-17A-producing CD4^+^CD44^+^CD90.2^+^ T cells was determined in single cell suspensions of lungs by flow cytometry after restimulation with anti-CD3/CD28. Representative density plots of IL-17A-producing CD4^+^CD44^+^CD90.2^+^ T cells on day 42 and frequencies of IL-17A-producing CD4^+^CD44^+^CD90.2^+^ T cells during the course of infection are shown. (**b**) CD4^+^ T cells were purified from lung cell suspensions by MACS and restimulated with ESAT-6_1-20_. The frequency of IL-17A-producing cells was determined by ELISPOT. (**c**) After reverse transcription of isolated lung RNA, gene expression of *Cxcl9*, *Cxcl10* and *Cxcl11* was quantified by real-time RT-PCR based on gene expression in uninfected mice. Mean values with standard deviations of 5 mice per group are shown. One representative experiment of two is shown. Data were statistically analyzed using the Mann–Whitney test defining differences between C57BL/6 and IL-6^−/−^ mice as significant (*, *p* ≤ 0.05; **, *p* ≤ 0.01).

The gp130 cytokine IL-6 acts as a proinflammatory cytokine facilitating cellular infiltration and is described to be required for the development and maintenance of a balanced TH17 immune response [16,17]. During Mtb infection, the absence of IL-6 resulted in a delayed cellular recruitment to lung tissue (Figure S1a). The relative number of IL-17A-producing cells within the CD90.2^+^CD4^+^CD44^+^ population was analyzed during the course of Mtb infection in IL-6^−/−^ mice by flow cytometry (Figure S2a; Figure 1a). In this context, the analysis pertained to polyclonal CD4^+^ T cells after restimulation with anti-CD3/CD28 for 4 hrs. As a negative control, cells were incubated in medium over the same time period. After 4 hrs of incubation in medium, the frequency of IL-17A-producing cells in lungs of C57BL/6 and IL-6^−/−^ mice were below approximately 0.7% (Figure S3a). In contrast, after restimulation with anti-CD3/CD28, the relative number of polyclonal IL-17A-expressing CD4^+^ T cells was highest in lung homogenates with approximately 4% in wild type and 3% in IL-6^−/−^ mice already 14 days after Mtb infection, declined until day 42 and increased at day 98 of experimental TB (Figure 1a). Although to a modest degree, the frequency of these polyclonal TH17 cells was significantly decreased in Mtb-infected IL-6^−/−^ mice. To gain a more precise picture of the actual impact of IL-6 on the Mtb-associated TH17 immune response, the frequency of ESAT6_1-20_-specific IL-17A-producing CD4^+^ T cells was measured by ELISPOT assay during the course of Mtb infection (Figure 1b). The relative number of these antigen-specific TH17 cells was almost constant over the entire course of infection in both groups but was significantly reduced in IL-6^−/−^ mice 98 days after Mtb infection.

Because IL-6 and IL-17A have a differential impact on the recruitment of CXCR3-expressing TH17 and TH1 cells [12,36], gene expression of *Cxcl9*, *Cxcl10* and *Cxcl11* in lung homogenates from Mtb-infected mice was examined by quantitative real-time RT-PCR (Figure 1c). The expression of these CXCR3 chemokines increased up to 42 days after infection with Mtb and remained at this level. While the expression of *Cxcl10* and *Cxcl11* in C57BL/6 and IL-6^−/−^ mice was similar, it was significantly increased for *Cxcl9* in IL-6^−/−^ mice on day 14 and tended to remain higher compared to wild type animals in the further course of infection. Together, these results indicate that IL-6 is not absolutely required for the development of a TH17 immune response and an associated CXCR3 chemokine induction during Mtb infection.

#### 3.1.2. The Regulatory Immune Response in IL-6^−/−^ Mice after Infection with Mtb

**Figure 2:**
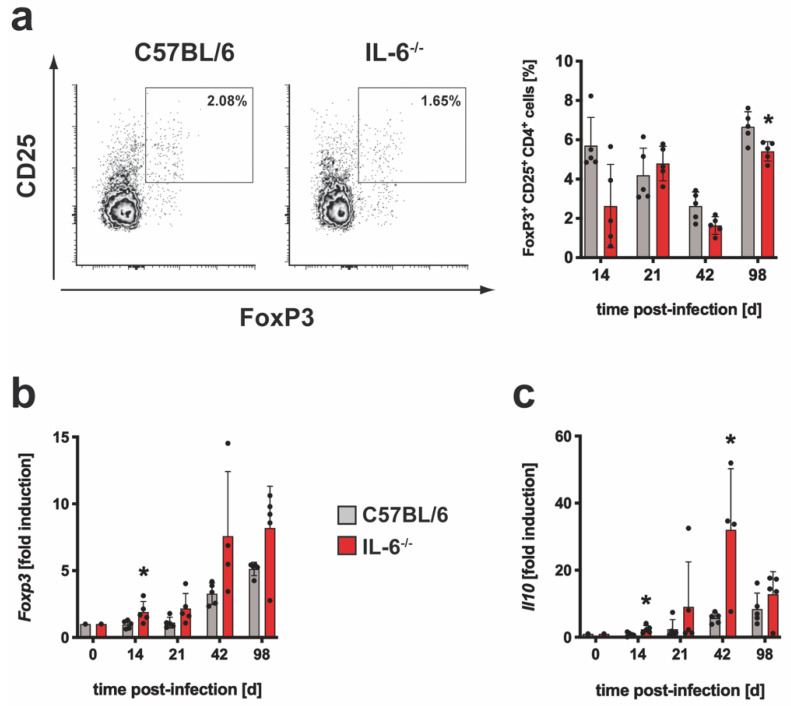
The regulatory immune response in Mtb-infected IL-6^−/−^ mice. C57BL/6 (grey symbols) and IL-6^−/−^ (red symbols) mice were infected with approximately 100 CFU Mtb via the aerosol route. At the indicated time points, lungs were removed and further processed. (**a**) The relative amount of the FoxP3^+^CD25^+^ population within the CD4^+^ T cells was determined in single cell suspensions of lungs by flow cytometry. Representative density plots of FoxP3^+^CD25^+^CD4^+^ T cells on day 42 and frequencies of FoxP3^+^CD25^+^CD4^+^ T cells during the course of infection are shown. (**b**,**c**) After reverse transcription of isolated lung RNA, gene expression of (**b**) *Foxp3* and (**c**) *Il10* was quantified by real-time RT-PCR based on gene expression in uninfected mice. Mean values with standard deviations of 5 mice per group are shown. One representative experiment of two is shown. Data were statistically analyzed using the Mann–Whitney test defining differences between C57BL/6 and IL-6^−/−^ mice as significant (*, *p* ≤ 0.05).

T_reg_ are modulators of the TH17 and TH1 immune response and IL-6 controls the induction of these cells [16]. By staining of Foxp3 (Figure S2b), it was determined whether the frequency of regulatory CD4^+^ T cells was modulated in Mtb-infected IL-6^−/−^ mice (Figure 2a). On day 98 post infection, the relative amount of Foxp3^+^ T_reg_ was slightly decreased whereas during the earlier progression of infection comparable frequencies of these cells could be detected in wild type and IL-6^−/−^ mice.

Because FoxP3 is involved in T_reg_ function, a potentially altered gene expression of *Foxp3* in the absence of IL-6 was monitored in Mtb-infected wild type and IL-6^−/−^ mice by quantitative real time RT-PCR (Figure 2b). *Foxp3* expression increased steadily in either group after infection but tended to be increased in IL-6^−/−^ mice and was significantly different on day 14 (Figure 2b). IL-10, produced by T cells, macrophages and other cells, also regulates cell-mediated immune responses during experimental TB [37,38]. *ll10* gene expression was significantly increased in IL-6^−/−^ mice on days 14 and 42 when compared to C57BL/6 mice (Figure 2c). Together, during experimental TB the absence of IL-6 appears to have no impact on the pulmonary infiltration and function of T_reg_ but rather promotes IL-10 production presumably by other cells.

#### 3.1.3. The Overall TH1 Immune Response and Subsequent Macrophage Effector Mechanism Are Not Impaired in Mtb-Infected IL-6^−/−^ Mice

**Figure 3:**
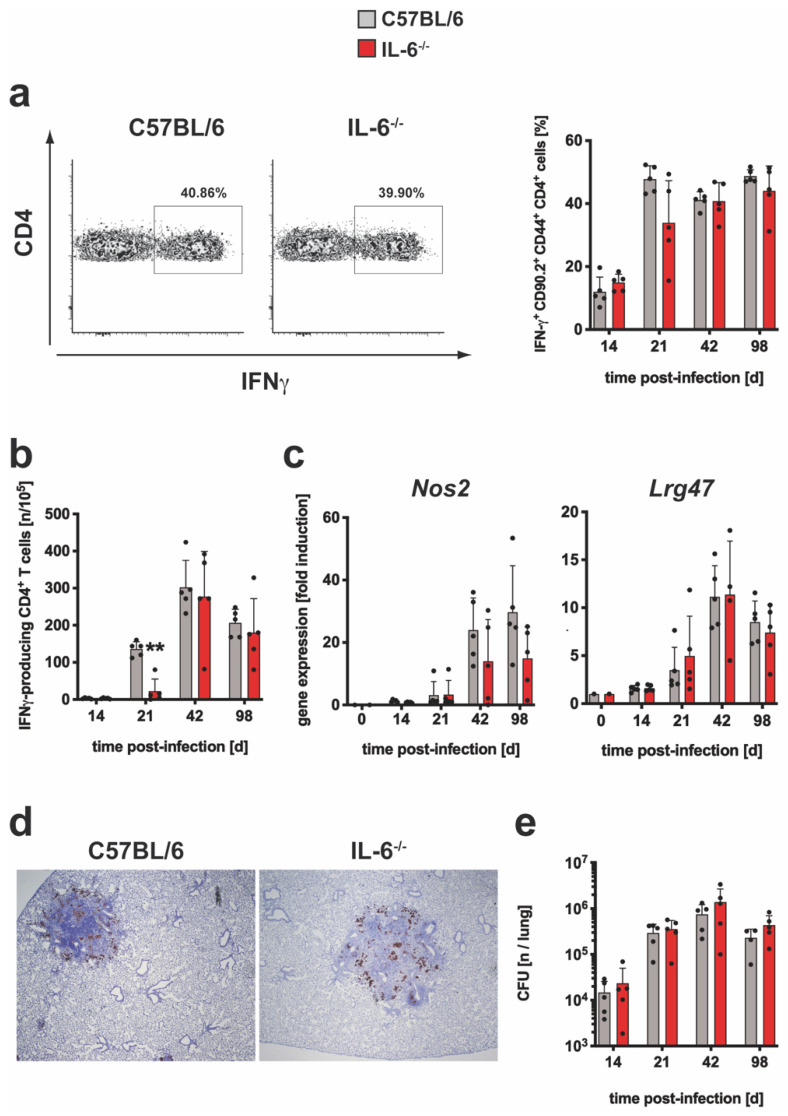
The TH1 immune response and macrophage effector functions in IL-6^−/−^ mice during experimental TB. C57BL/6 (grey symbols) and IL-6^−/−^ (red symbols) mice were infected with approximately 100 CFU Mtb via the aerosol route. At the indicated time points, lungs were removed and further processed. (**a**) The relative amount of IFNγ-producing CD4^+^CD44^+^CD90.2^+^ T cells was determined in single cell suspensions of lungs by flow cytometry after restimulation with anti-CD3/CD28. Representative density plot of IFNγ-producing CD4^+^CD44^+^CD90.2^+^ T cells on day 42 and IFNγ-producing CD4^+^CD44^+^CD90.2^+^ T cells during the course of infection are shown. (**b**) CD4^+^ T cells were purified from lung cell suspensions by MACS and restimulated with ESAT-6_1-20_. The frequency of IFN γ-producing cells was determined by ELISPOT. (**c**) Gene expression of *Nos2* and *Lrg47* was quantified by real-time RT-PCR based on gene expression in uninfected mice. (**d**) Representative immunohistochemical staining of NOS2 in lung sections of experimental mice 42 days of infection. (**e**) At the indicated time points, bacterial loads in the lungs were determined. Mean values with standard deviations of 5 mice per group are shown. One representative experiment of two is shown. Data were statistically analyzed using the Mann–Whitney test defining differences between C57BL/6 and IL-6^−/−^ mice as significant (**, *p* ≤ 0.01).

To elucidate whether the development of a TH1 immune response is affected in Mtb-infected IL-6^−/−^ mice, the frequency of IFNγ-producing C90.2^+^CD4^+^CD44^+^ T cells was analyzed during experimental TB by flow cytometry (Figure S2a; Figure 3a). At different time points of infection, single lung cell suspensions were prepared, and intracellular staining of IFNγ in CD4^+^ T cells was performed after 4 hrs of incubation in medium (Figure S3a) or after restimulation with anti-CD3/CD28 (Figure 3a). The frequency of IFNγ-secreting cells without restimulation was approximately 2% (Figure S3a). The proportion of TH1 cells increased after polyclonal restimulation in the course of experimental TB to approximately 40%. However, no significant differences between wild type and IL-6^−/−^ mice were detected. To evaluate the antigen-specific TH1 immune response, the frequencies of ESAT6_1-20_-specific IFNγ-producing CD4^+^ T cells were measured by ELISPOT assays at different time points of infection (Figure 3b). Although the frequency of IFNγ-producing CD4^+^ T cells was significantly reduced in IL-6^−/−^ mice 21 days after Mtb infection, comparable amounts of IFNγ-secreting CD4^+^ T cells could be detected during the following course of infection.

Since the effector functions mediated by IFNγ in macrophages play a key role in the control of an Mtb infection [39,40], the gene expression of the effector molecules *Nos2* and *Lrg47* in lung homogenates of infected animals was measured (Figure 3c). Both genes were induced in either group on day 21 and gene expression of *Lrg47* peaked on day 42. Although no significant differences between wild type and IL-6^−/−^ mice were found at any time point, the gene expression of *Nos2* tended to be reduced in the absence of IL-6 on days 42 and 98. In order to analyze macrophage activation in the course of granuloma formation after infection with Mtb, NOS2 was examined immunohistochemically in lung tissue sections of infected mice (Figure 3d). Basically, the granuloma structure in C57BL/6 and IL-6^−/−^ mice was comparable and the expression of NOS2 in the lesions of both groups was also similar.

To eventually evaluate effects of IL-6 on the outcome of Mtb infection, bacterial loads were determined in lungs (Figure 3e), spleen (Figure S4a) and liver (Figure S4a) at different time points of infection. After aerosol infection, CFU in all of these organs rose sharply until reaching a plateau on day 42. During the whole course of infection, the bacterial load in the lungs, spleen and liver of IL-6^−/−^ mice was comparable to C57BL/6 mice. Hence, the deletion of IL-6 had no influence on the outcome of an aerosol infection with Mtb.

### 3.2. Experimental TB in CD4^cre^; gp130^loxP/loxP^ Mice

#### 3.2.1. Gene Expression of *Il12b*, *Tnf* and *Il27* was Decreased Whereas mRNA Levels of *Il6* were Elevated in Mtb-Infected CD4^cre^; gp130^loxP/loxP^ Mice

Based on the so far presented results, IL-6 appears to only moderately promote or regulate TH17, TH1 and regulatory immune response after Mtb infection, respectively. Finally, IL-6 is dispensable for the development of a protective immune response during experimental TB. Because IL-6 is a pleiotropic cytokine that modulates pro- and anti-inflammatory responses and acts on innate and adaptive immune cells as well as of non-immune cells through classical and trans-signaling, in the global IL-6^−/−^ mouse model, the cell-specific functions of this gp130 cytokine may be concealed.

**Figure 4:**
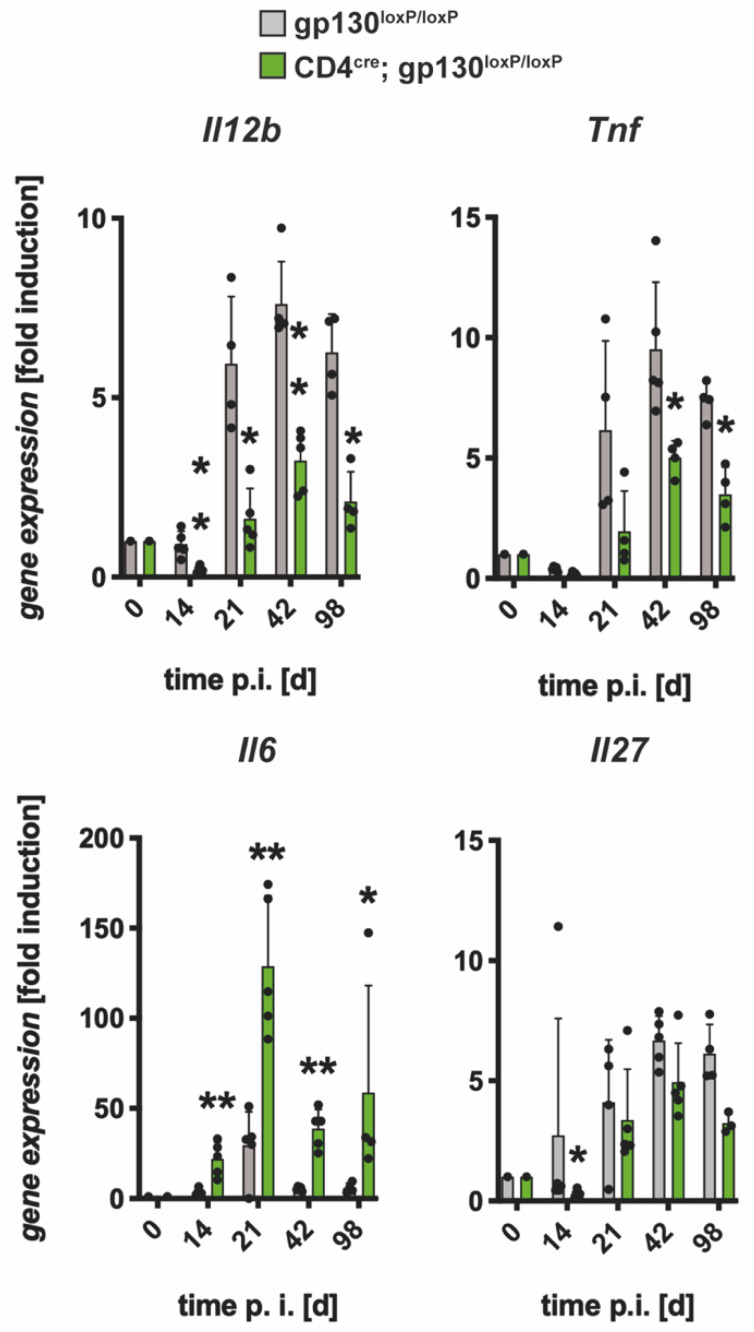
Cytokine gene expression in the lungs of Mtb-infected CD4^cre^; gp130^loxP/loxP^ mice. gp130^loxP/loxP^ (grey symbols) and CD4^cre^; gp130^loxP/loxP^ mice (green symbols) were infected with approximately 100 CFU Mtb via the aerosol route. Lung RNA was isolated at the indicated time points. After reverse transcription, expression of *Il12b*, *Tnf*, *Il6* and *Il27* was quantified by real-time RT-PCR based on gene expression in uninfected mice. Mean values with standard deviations of 5 mice per group are shown. One representative experiment of two is shown. Data were statistically analyzed using the Mann–Whitney test defining differences between cre-negative littermates and CD4^cre^; gp130^loxP/loxP^ mice as significant (*, *p* ≤ 0.05; **, *p* ≤ 0.01).

To characterize the immune response of T cell-specific gp130-deficient (CD4^cre^; gp130^loxP/loxP^) mice after Mtb infection on the gene expression level, the induction of the cytokines *Il12b*, *Tnf, Il6* and *Il27* in lung homogenates was measured by quantitative real time RT-PCR (Figure 4).

After aerosol infection, gene expression of *Il12b*, *Tnf* and *Il27* increased until day 42. When compared to cre-negative littermates, gene expression of *Il12b* and *Tnf* was significantly reduced in CD4cre; gp130^loxP/loxP^ mice from day 21 on. The reduced *Il27* gene expression in CD4^cre^gp130^f/f^ mice was only significantly different on day 14. In striking contrast, gene expression of *Il6* was very strongly increased at all time points with a significant difference. Hence, the absence of gp130 on T cells results in an overall disturbed expression of inflammatory and regulatory cytokines during experimental TB.

#### 3.2.2. The TH17 Immune Response in Mtb-Infected CD4^cre^; gp130^loxP/loxP^ Mice Was Only Moderately Affected

**Figure 5:**
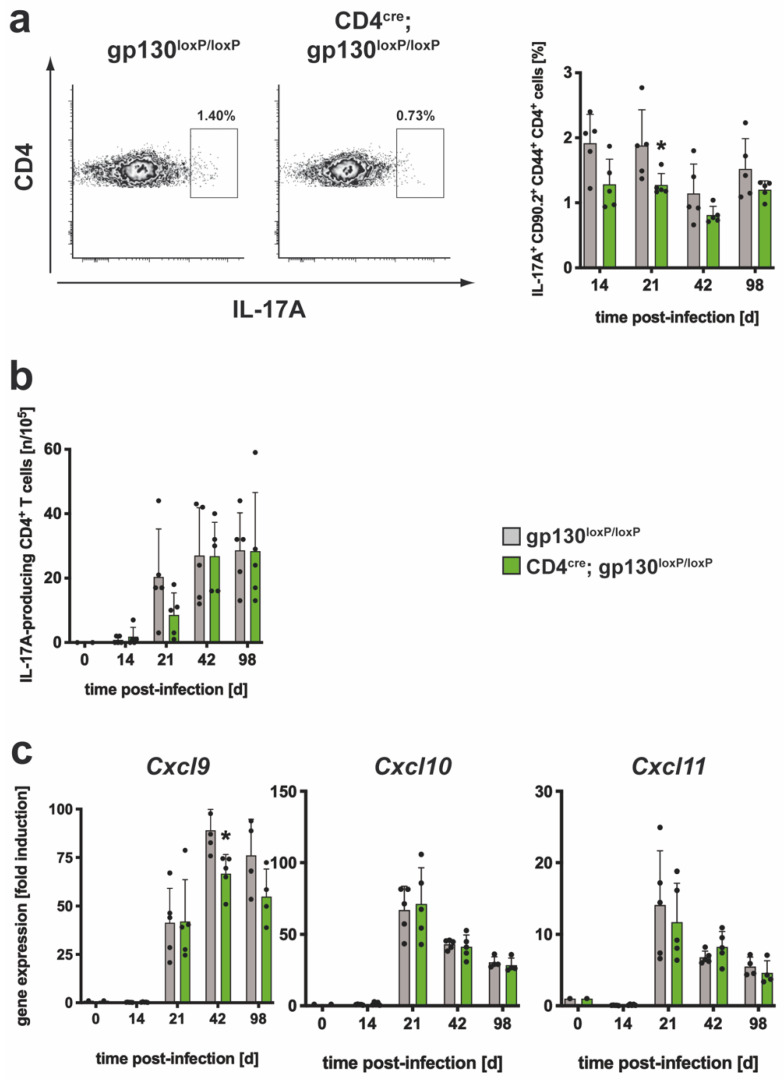
The TH17 immune response in CD4^cre^; gp130^loxP/loxP^ mice during experimental TB. gp130^loxP/loxP^ (grey symbols) and CD4^cre^; gp130^loxP/loxP^ mice (green symbols) were infected with approximately 100 CFU Mtb via the aerosol route. At the indicated time points, lungs were removed and further processed. (**a**) The relative amount of IL-17A-producing CD4^+^CD44^+^CD90.2^+^ T cells was determined in single cell suspensions of lungs by flow cytometry after restimulation with anti-CD3/CD28. Representative density plots of IL-17A-producing CD4^+^CD44^+^CD90.2^+^ T cells on day 42 and frequencies of IL-17A-producing CD4^+^CD44^+^CD90.2^+^ T cells during the course of infection are shown. (**b**) CD4^+^ T cells were purified from lung cell suspensions by MACS and restimulated with ESAT-6_1-20_. The frequency of IL-17A-producing cells was determined by ELISPOT. (**c**) After reverse transcription of isolated lung RNA, gene expression of *Cxcl9*, *Cxcl10* and *Cxcl11* was quantified by real-time RT-PCR based on gene expression in uninfected mice. Mean values with standard deviations of 5 mice per group are shown. One representative experiment of two is shown. Data were statistically analyzed using the Mann–Whitney test defining differences between cre-negative littermates and CD4^cre^; gp130^loxP/loxP^ mice as significant (*, *p* ≤ 0.05).

To follow a potentially altered TH17 immune response, the frequency of IL-17A-producing CD4^+^ T cells (Figure 5a,b) and the gene expression of CXCR3 chemokines (Figure 5c) was analyzed in gp130^loxP/loxP^ and CD4^cre^; gp130^loxP/loxP^ mice after Mtb infection. During experimental TB, the cellular infiltration of lungs similarly increased in both groups (Figure S1b). The frequency of polyclonal IL-17A-producing cells within the CD90.2^+^CD4^+^CD44^+^ population was analyzed during the course of Mtb infection by flow cytometry (Figure S2a). After 4 hrs of incubation in medium, the background frequency of IL-17A-secreting cells in lungs of both groups was below approximately 0.3% (Figure S3b). After restimulation with anti-CD3/CD28, the frequency of polyclonal TH17 cells was already on maximum at day 14 in both groups and only decreased slowly until the end of the experiment (Figure 5a). Although, the relative amount of polyclonal IL-17A-producing CD4^+^ T cells was slightly reduced in CD4^cre^; gp130^loxP/loxP^ mice during the whole course of infection, the difference to cre-negative control animals was only significant on day 21. To evaluate the antigen-specific TH17 immune response after infection with Mtb, the frequency of ESAT6_1-20_-specific IL-17A-producing CD4^+^ T cells was measured by ELISPOT assay (Figure 5b). The relative amount of antigen-specific TH17 cells increased steadily in the course of the infection in both groups. Although not significantly altered, on day 21 after Mtb infection, a lower frequency of antigen-specific IL-17A-expressing CD4^+^ T cells could be observed in CD4^cre^; gp130^loxP/loxP^ mice. At all other time points of infection, a comparable frequency of antigen-specific TH17 cells could be measured in both cre-negative littermates and CD4^cre^; gp130 ^loxP/loxP^ mice. To evaluate the effect of a T cell-specific gp130 deficiency on the gene expression of the CXCR3 chemokines *Cxcl*9, *Cxcl10* and *Cxcl11* in lung homogenates from Mtb-infected mice, a quantitative real-time RT-PCR was carried out (Figure 5c). *Cxcl9* gene expression was induced after Mtb infection and was found to peak on day 42. When compared to cre-negative littermates, the *Cxcl9* mRNA level was only significantly decreased in CD4^cre^; gp130^loxP/loxP^ mice on day 42. The expression of *Cxcl10* and *Cxcl11* was maximal in both groups on day 21 after infection with Mtb, after which it decreased slowly and was not significantly different at any time point. Overall, these results indicate an efficient induction of a TH17 immune response during experimental TB in the absence of gp130-mediated signaling in T cells.

#### 3.2.3. The Regulatory Immune Response in Mtb-Infected CD4^cre^; gp130^loxP/loxP^ Mice

**Figure 6:**
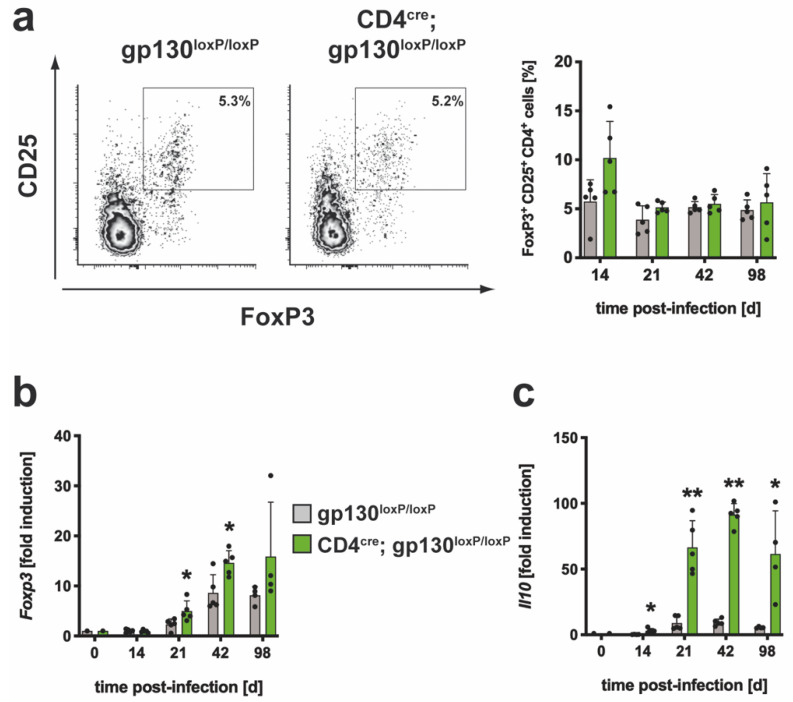
The regulatory immune response in Mtb-infected CD4^cre^; gp130^loxP/loxP^ mice. gp130^loxP/loxP^ (grey symbols) and CD4^cre^; gp130^loxP/loxP^ mice (green symbols) were infected with approximately 100 CFU Mtb via the aerosol route. At the indicated time points, lungs were removed and further processed. (**a**) The relative amount of the FoxP3^+^CD25^+^ population within the CD4^+^ T cells was determined in single cell suspensions of lungs by flow cytometry. Representative density plots of FoxP3^+^CD25^+^CD4^+^ T cells on day 42 and frequencies of FoxP3^+^CD25^+^CD4^+^ T cells during the course of infection are shown. (**b**,**c**) After reverse transcription of isolated lung RNA, gene expression of (**b**) *Foxp3* and (**c**) *Il10* was quantified by real-time RT-PCR based on gene expression in uninfected mice. Mean values with standard deviations of 5 mice per group are shown. One representative experiment of two is shown. Data were statistically analyzed using the Mann–Whitney test defining differences between cre-negative littermates and CD4^cre^; gp130^loxP/loxP^ mice as significant (*, *p* ≤ 0.05; **, *p* ≤ 0.01).

After intracellular staining of Foxp3 in CD25^+^CD4^+^ T cells, it was determined whether the frequency of T_reg_ was modulated in Mtb-infected CD4^cre^; gp130^loxP/loxP^ mice (Figure S2b; Figure 6). At day 14 post infection, the relative amount of T_reg_ was tendentially higher in CD4^cre^; gp130^loxP/loxP^ mice. Over the further course of infection, the frequencies of Foxp3^+^CD25^+^CD4^+^ T cells were comparable in cre-negative littermates and CD4^cre^; gp130^loxP/loxP^ mice. A potentially modulated gene expression of *Foxp3* and *Il10* in the absence of gp130 on T cells was evaluated in Mtb-infected cre-negative and CD4^cre^; gp130^loxP/loxP^ mice by quantitative real time RT-PCR (Figure 6b,c). The expression of both genes increased steadily in either group after infection. In contrast to the constant level of Foxp3^+^ T_reg_ at the later phase of infection, the induction of *Foxp3* was upregulated in CD4^cre^; gp130^loxP/loxP^ mice (Figure 6b). Because *Il10* gene expression was also highly elevated in cre-positive mice from 21 days, the absence of gp130 on T cells resulted in an enhanced activation of a regulatory immune response during experimental TB. Together, immune regulation during experimental TB appears to be disturbed in the absence of gp130 on T cells.

#### 3.2.4. After Mtb Infection, The TH1 Immune Response Was Not Affected by the Absence of gp130 on T Cells yet TH1-Mediated Macrophage Effector Responses Were Severely Impaired

**Figure 7:**
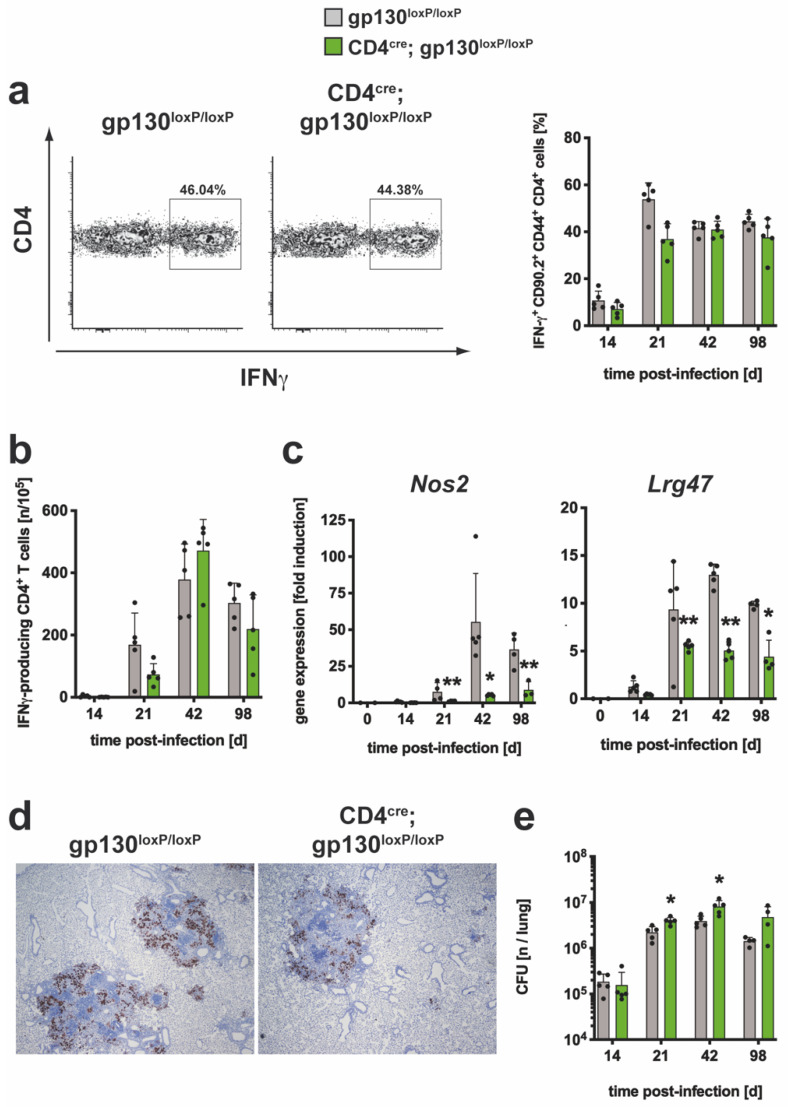
The TH1 immune response in CD4^cre^; gp130^loxP/loxP^ mice during experimental TB. gp130^loxP/loxP^ (grey symbols) and CD4^cre^; gp130^loxP/loxP^ mice (green symbols) were infected with approximately 100 CFU Mtb via the aerosol route. At the indicated time points, lungs were removed and further processed. (**a**) The relative amount of IFNγ-producing CD4^+^CD44^+^CD90.2^+^ T cells was determined in single cell suspensions of lungs by flow cytometry after restimulation with anti-CD3/CD28. Representative density plots of IFNγ-producing CD4^+^CD44^+^CD90.2^+^ T cells on day 42 and IFNγ-producing CD4^+^CD44^+^CD90.2^+^ T cells during the course of infection are shown. (**b**) CD4^+^ T cells were purified from lung cell suspensions by MACS and restimulated with ESAT-6_1-20_. The frequency of IFNγ-producing cells was determined by ELISPOT. (**c**,**d**) After reverse transcription of isolated lung RNA, gene expression of (**c**) *Nos2* and *Lrg47* was quantified by real-time RT-PCR based on gene expression in uninfected mice. (**d**) Representative immunohistochemical staining of NOS2 in lung section of experimental mice 42 days of infection. (**e**) At the indicated time points, bacterial loads in the lungs were determined. Mean values with standard deviations of 5 mice per group are shown. One representative experiment of two is shown. Data were statistically analyzed using the Mann–Whitney test defining differences between cre-negative littermates and CD4^cre^; gp130^loxP/loxP^ mice as significant (*, *p* ≤ 0.05; **, *p* ≤ 0.01).

To elucidate whether the T cell-specific deletion of gp130 has a direct effect in favoring a potential modulated TH1 immune response in Mtb-infected CD4^cre^; gp130^loxP/loxP^ mice, the frequency of IFNγ-expressing CD90^+^CD4^+^CD44^+^ T cells was measured (Figure S2a; Figure 7a).

Single lung cell suspensions were prepared and flow cytometric analysis after intracellular staining of IFNγ in CD4^+^ T cells and 4 hrs of incubation in medium revealed a background frequency of approximately 2% (Figure S3b). After restimulation with anti-CD3/CD28, the frequency of IFNγ-producing CD4 T cells increased to 50% on day 21 and stayed at this level until the end of the experiment (Figure 7a). However, during the course of infection, the frequency of IFNγ-secreting CD4^+^ T cells was similar in both groups. To evaluate the antigen-specific TH1 immune response, the relative proportion of ESAT6_1-20_-specific IFNγ-producing CD4^+^ T cells was measured by ELISPOT assay at different time points of infection (Figure 7b). The frequencies of antigen-specific TH1 cells increased after Mtb infection until day 42 after which the numbers fell slightly. When compared to cre-negative littermates, no significant differences in the in the frequency of IFNγ-producing CD4^+^ T cells were found in CD4^cre^; gp130^loxP/loxP^ mice.

Despite the virtually unaltered IFNγ-dependent TH1 immune response in Mtb-infected CD4^cre^; gp130^loxP/loxP^ mice, the induction of the IFNγ downstream macrophage effector molecules NOS2 and LRG47 was further analyzed. Except during the very early phase at day 14 after infection, the gene expression of *Nos2* and *Lrg47* was significantly reduced in lung homogenates of CD4^cre^; gp130^loxP/loxP^ mice during the further course of infection (Figure 7c). This was confirmed by immunohistochemical staining of NOS2 in lung sections of Mtb-infected mice, in which the expression of NOS2 in the lesions of gp130-deficient animals was again lower (Figure 7d). Since, in contrast to Mtb-infected IL-6^−/−^ mice, both cytokine expression and macrophage activation were strongly modulated compared to the infected control mice, histopathological investigations of lung sections were carried out during the course of the experimental TB (Figure S5). However, no differences in tissue inflammation between gp130^loxP/loxP^ and CD4^cre^; gp130^loxp/loxP^ mice were observed. Together, the absence of gp130 on T cells had no direct effect on the development of a TH1 immune response during experimental TB. Apparently, however, the loss of T cell gp130 had an indirect suppressive effect on the expression of effector mechanisms in macrophages.

To investigate the impact of gp130 on T cells on the outcome of Mtb infection, bacterial loads were determined in lungs (Figure 7e), spleen (Figure S4b) and liver (Figure S4b) at different time points of infection. After aerosol infection, the CFU in all of these organs increased until reaching a plateau on day 42. During the whole course of infection, the bacterial load in the spleen and liver of gp130^loxP/loxP^ and CD4^cre^; gp130 ^loxP/loxP^ mice was comparable (Figure S4b). However, on day 21 and 42 of Mtb infection the bacterial load in the lungs of CD4^cre^; gp130 ^loxP/loxP^ mice was moderately but significantly increased when compared to cre-negative littermates (Figure 7e). In summary, the absence of gp130 on T cells has no direct effect on the development of TH1 cells, but the indirect impairment of macrophage activation tended to result in less control of bacterial growth.

## 4. Discussion

In the past decades, IL-6 has been proposed as a major contributor to the generation of TH17 immune responses in numerous studies [16,17,41,42,43]. Together with TGF-β, IL-6 in vitro promotes the differentiation of TH17 cells from naïve T cells and simultaneously suppresses the generation of T_reg_ [16,17]_._ In murine models of autoimmune diseases, IL-6 mediates the induction of pathogenic TH17 cells to eventually trigger the onset of disease [41,42]. Moreover, following intranasal infection with Group A streptococcus, IL-6^−/−^ mice are unable to generate a protective TH17 immune response, indicating that IL-6 is a crucial cytokine for TH17 differentiation also during bacterial infections [43]. In contrast to these findings, the present study, however, demonstrates that during experimental TB, the gp130-dependent cytokine IL-6 has a small direct impact for the development of TH17 cells.

During infection with Mtb, the major TH17 cytokine IL-17A mediates the formation of granuloma via chemokine induction [11,44] and the accumulation of a protective TH1 immune response, but on the other hand, it contributes to the early neutrophil-dependent inflammation [45]. Whereas the modest expression of IL-17A in the context of a low-dose infection with Mtb H37Rv does not impair mycobacterial burdens [11], the proinflammatory cytokine appears to contribute to protection against Mtb under conditions of IL-17A overproduction [11,12,15]. However, it also provokes immunopathology caused by excessive inflammation [15]. Thus, a controlled induction of IL-17A may represent a promising strategy in the context of vaccination or host-directed therapy approaches against TB. Here, we investigated the impact of IL-6 for the development of a TH17 immune response after infection with Mtb by use of IL-6^−/−^ mice. On account of the pleotropic nature of IL-6 [32], we additionally analyzed the cellular immune response during the course of experimental TB in mice with a T cell specific gp130 deficiency.

Mtb-infected IL-6^−/−^ mice, as the present study demonstrates, exhibit a reduced polyclonal TH17 immune response over the entire course of infection. Antigen-specific TH17 cells were still generated in IL-6^−/−^ mice after infection with Mtb, although their frequency again tended to be reduced when compared to C57BL/6 mice. Although the reason for the here observed variations between the frequencies of polyclonal and antigen-specific TH17 cells during the course of Mtb infection remains open, it has to be stated that the latter T cell population does not comprise all Mtb antigen-specific TH17 cells but only those directed against the ESAT6_1-20_ peptide. Polyclonal TH17 cells, on the other hand, can be expected to comprise the entire TH17 population in the infected lung, including also non Mtb-specific cells. Nevertheless, altogether, the analysis of ESAT6_1-20_-specific CD4^+^ T cells may depict the actual impact of IL-6 on the induction of the Mtb-associated TH17 immune response more precisely than evaluation of polyclonally stimulated cells. In summary, these data indicate that IL-6 is not absolutely required for the initiation and maintenance of TH17 cells in the context of experimental TB.

After induction of EAE, IL-6-mediated signaling is crucial for the disease-related inhibition of T_reg_ conversion from naïve T cells [41]. Moreover, in this context, IL-6^−/−^ mice are able to mount a TH17 immune response only in the absence of T_reg_. In the present study, however, both the Mtb-induced pulmonary infiltration and the function of T_reg_ was not affected by the absence of IL-6. Here, other TH17-driving factors may rather ensure TH17 induction in the Mtb-infected IL-6^−/−^ mice. As IL-6 mediates the differentiation of TH17 cells in a Signal Transducer and Activator of Transcription (STAT)3-dependent manner [46], particularly other STAT3-activating cytokines may compensate for IL-6 during TH17 polarization. The STAT3-inducer IL-21 [47] indeed represents a crucial initiator of TH17 development under inflammatory conditions in mice and men [48,49,50,51]. During experimental TB, the cytokine contributes to optimal control of Mtb [52,53], accompanied by enhanced expression levels of *Il17a* [52]. However, IL-21 appeared to be dispensable for the induction of a vaccine-associated protective TH17 immune response after Mtb challenge [54]. Of great importance for the maintenance of a TH17 immune response during primary TB [29,30] and after vaccination [12] is the STAT3-activating cytokine IL-23 [55]. During vaccination against Mtb, IL-23, however, also appeared not to be involved in the initial priming of TH17 cells [12]. Hence, future studies may shed more light on the cytokine environment and cellular composition necessary for the differentiation of TH17 cells during infection with Mtb.

To examine whether the moderate modulation of TH17 cells in the absence of IL-6 is associated with an impaired protective immune response against Mtb, we further analyzed the integrity of TH1 immunity and the bacterial loads during the course of infection. Whereas the induction of TH1 cells during Mtb infection was only weakly affected in IL-6^−/−^ mice, levels of *Nos2* were tendentially reduced as a result of IL-6 deficiency. Nonetheless, the outcome of Mtb infection in IL-6^−/−^ and C57BL/6 mice was comparable. These observations are line with previous findings, which together indicate that IL-6 has a rather small overall impact on the protective immune response in TB [56,57,58]. As during Mtb infection, the pleiotropic cytokine differentially mediates pro and anti-inflammatory functions depending on the target cell, differential effects of IL-6 may be neutralized within the analysis of IL-6^−/−^ mice. To overcome this limitation of the IL-6^−/−^ mouse model, the additional analysis of mice with a cell-type specific deficiency of gp130-mediated signaling provides a beneficial tool.

Within the present study, the outcome of Mtb infection was investigated in mice with a T cell-specific deficiency of gp130 for the first time. In the context of autoimmunity [41] or nematode infection [59], these CD4^cre^; gp130^loxP/loxP^ mice exhibit abrogated TH1 and TH17 immune responses. Here, we demonstrate that during Mtb infection, the T cell specific deficiency of gp130-mediated signaling results in a differentially modulated expression of proinflammatory and immunosuppressive cytokines. Nevertheless, the absence of T cell gp130 had a minor overall impact on both the TH1 and TH17 immune response but also on the accumulation of T_reg_. Whereas in Mtb-infected IL-6^−/−^ mice at least the frequencies of polyclonal TH17 cells were significantly reduced over the entire course of infection, the T cell specific deficiency of gp130-mediated signaling resulted in a decreased TH17 immune response on both the polyclonal and antigen-specific level only the early phase of infection. This discrepancy between the degree of TH17 cell induction in IL-6^−/−^ and CD4^cre^; gp130^loxP/loxP^ mice might be explained with the additional lack of signaling by other gp130-dependent cytokines in the latter mouse model. In particular, the IL-12 family cytokine IL-27 regulates TH17 immune responses during experimental TB and thereby inhibits optimal mycobacterial containment [15]. Nevertheless, these results indicate that, whereas IL-23 represents a key factor for the maintenance of TH17 immune responses during experimental TB [29,30], IL-6 rather exerts a TH17-promoting influence during the initial phase of T cell activation.

Of note, specifically on the antigen-specific level, development of the TH17 immune responses differed between both ELISPOT experiments shown in the present study. Whereas in C57BL/6 wild type and IL-6^−/−^ mice the frequency of antigen-specific TH17 cells was almost constant over the entire course of infection, both cre-negative littermates and CD4^cre^; gp130^loxP/loxP^ mice exhibited steadily increasing relative amounts of these cells during the course of the infection. It remains open, whether this general discrepancy constitutes a biological or technical deviation.

Together, these findings further support the hypothesis that IL-6, as a major gp130-dependent cytokine, does not represent a critical factor for the development of TH17 immunity during Mtb infection. In light of the findings presented here, it seems surprising that in human patients the administration of the mIL-6Rα-neutralizing drug tocilizumab—which is used for the treatment of chronic inflammatory diseases—is connected to an increased risk for reactivation of TB [60]. However, it additionally has to be considered that during murine TB the overall impact of IL-6 appears to depend on experimental conditions such as the bacterial dose and the route of infection [31,56,61]. Therefore, it may be possible that IL-6 exerts a more significant influence on the development of a TH17 immune response during a high-dose infection with the lab-adapted Mtb strain H37rv. A similar effect might also arise from experimental infection with other more virulent Mtb strains or clinical isolates.

Notably, in contrast to the unaffected TH1 immune response, Mtb-infected CD4^cre^; gp130^loxP/loxP^ mice show strongly reduced gene expression levels of *Nos2* and *Lrg47* and NOS2 production in granulomatous lesions when compared to cre-negative littermates. As the induction of those antimicrobial effector molecules clearly depends on IFNγ [62,63], this finding appears rather surprising. However, a probable explanation may be provided by the enhanced expression of *Il6* in the T cell-specific gp130-deficient mice. As we have demonstrated previously, IL-6 specifically exerts a suppressive effect on the expression of inflammatory cytokines and effector molecules on macrophages [57]. Given the suppressive impact of the regulatory cytokine IL-10 in TB [64], the strongly elevated expression of *Il10* observed in the Mtb-infected CD4^cre^; gp130^loxP/loxP^ mice may also contribute to the inhibition of effector molecules. In accordance with the reduced induction of macrophage effector functions, bacterial containment in the lung was slightly impaired in the absence of gp130 on T cells. It is important to note here that it remains unclear, whether the elevated expression levels of *Il6* and *Il10* in the Mtb-infected CD4^cre^; gp130^loxP/loxP^ mice indeed go back to T cells or constitute an indirect effect of the impaired T cell functionality in those mice. While monocytes and macrophages appear to be the most important sources of IL-6 in TB [60,65], IL-10 seems to be produced by varying cellular sources of both the innate and adaptive immune system during the course of Mtb infection [64] and has fundamental suppressive effects on antimycobacterial effector responses in macrophages [37,38]. Future studies will be necessary to further elucidate a potential direct regulatory impact of T cell-secreted cytokines on the activation of Mtb-infected macrophages and the subsequent increase in mycobacterial growth.

## 5. Conclusions

In summary, by use of complementary mouse models, the present study strongly suggests that IL-6 plays a subordinate role for the generation of TH17 immune responses during experimental TB—a finding that contrasts with the key function in TH17 development that has been ascribed to the cytokine in several other models [41,42,43]. As a targeted induction of IL-17A may represent an encouraging approach for improving vaccination or adjunct therapy against TB, identification of the critical TH17-driving factors during Mtb infection remains an important issue.

## Supplementary Materials

The following are available online at https://www.mdpi.com/2073-4409/10/1/9/s1, Figure S1: The number of lung cells during experimental TB. Figure S2: Gating strategies for intracellular detection of IFNγ, IL-17A and FoxP3^+^ by flow cytometry. Figure S3: The TH1/TH17 immune response during experimental TB—gating of unstimulated cells. Figure S4: Bacterial loads in spleen and liver after infection with Mtb. Figure S5: Histopathological changes in lungs of gp130^loxP/loxP^ and CD4^cre^; gp130^loxP/loxP^ mice after infection with Mtb.

## References

[1] WHO (2019). Global Tuberculosis Report 2019.

[2] Maniar J.K., Kamath R.R., Mandalia S., Shah K., Maniar A. (2006). HIV and tuberculosis: Partners in crime. Indian J. Dermatol. Venereol. Leprol..

[3] Tufariello J.M., Chan J., Flynn J.L. (2003). Latent tuberculosis: Mechanisms of host and bacillus that contribute to persistent infection. Lancet Infect. Dis..

[4] Keane J., Gershon S., Wise R.P., Mirabile-Levens E., Kasznica J., Schwieterman W.D., Siegel J.N., Braun M.M. (2001). Tuberculosis associated with infliximab, a tumor necrosis factor alpha-neutralizing agent. N. Engl. J. Med..

[5] Mohan V.P., Scanga C.A., Yu K., Scott H.M., Tanaka K.E., Tsang E., Tsai M.M., Flynn J.L., Chan J. (2001). Effects of tumor necrosis factor alpha on host immune response in chronic persistent tuberculosis: Possible role for limiting pathology. Infect. Immun..

[6] North R.J., Jung Y.-J. (2004). Immunity to tuberculosis. Annu. Rev. Immunol..

[7] Mayer-Barber K.D., Barber D.L. (2015). Innate and Adaptive Cellular Immune Responses to Mycobacterium tuberculosis Infection. Cold Spring Harb. Perspect. Med..

[8] Cooper A.M., Dalton D.K., Stewart T.A., Griffin J.P., Russell D.G., Orme I.M. (1993). Disseminated tuberculosis in interferon gamma gene-disrupted mice. J. Exp. Med..

[9] Flynn J.L., Goldstein M.M., Chan J., Triebold K.J., Pfeffer K., Lowenstein C.J., Schreiber R., Mak T.W., Bloom B.R. (1995). Tumor necrosis factor-alpha is required in the protective immune response against Mycobacterium tuberculosis in mice. Immunity.

[10] Korbel D.S., Schneider B.E., Schaible U.E. (2008). Innate immunity in tuberculosis: Myths and truth. Microbes Infect./Inst. Pasteur.

[11] Gopal R., Monin L., Slight S., Uche U., Blanchard E., Junecko B.A.F., Ramos-Payan R., Stallings C.L., Reinhart T.A., Kolls J.K. (2014). Unexpected Role for IL-17 in Protective Immunity against Hypervirulent Mycobacterium tuberculosis HN878 Infection. PLoS Pathog..

[12] Khader S.A., Bell G.K., Pearl J.E., Fountain J.J., Rangel-Moreno J., Cilley G.E., Shen F., Eaton S.M., Gaffen S.L., Swain S.L. (2007). IL-23 and IL-17 in the establishment of protective pulmonary CD4^(+)^ T cell responses after vaccination and during Mycobacterium tuberculosis challenge. Nat. Immunol..

[13] Lindenstrom T., Woodworth J., Dietrich J., Aagaard C., Andersen P., Agger E.M. (2012). Vaccine-induced th17 cells are maintained long-term postvaccination as a distinct and phenotypically stable memory subset. Infect. Immun..

[14] Desel C., Dorhoi A., Bandermann S., Grode L., Eisele B., Kaufmann S.H. (2011). Recombinant BCG DeltaureC hly^+^ induces superior protection over parental BCG by stimulating a balanced combination of type 1 and type 17 cytokine responses. J. Infect. Dis..

[15] Erdmann H., Behrends J., Ritter K., Holscher A., Volz J., Rosenkrands I., Holscher C. (2018). The increased protection and pathology in Mycobacterium tuberculosis-infected IL-27R-alpha-deficient mice is supported by IL-17A and is associated with the IL-17A-induced expansion of multifunctional T cells. Mucosal Immunol..

[16] Bettelli E., Carrier Y., Gao W., Korn T., Strom T.B., Oukka M., Weiner H.L., Kuchroo V.K. (2006). Reciprocal developmental pathways for the generation of pathogenic effector TH17 and regulatory T cells. Nature.

[17] Veldhoen M., Hocking R.J., Atkins C.J., Locksley R.M., Stockinger B. (2006). TGFbeta in the context of an inflammatory cytokine milieu supports de novo differentiation of IL-17-producing T cells. Immunity..

[18] Aggarwal S., Ghilardi N., Xie M.H., de Sauvage F.J., Gurney A.L. (2003). Interleukin-23 promotes a distinct CD4 T cell activation state characterized by the production of interleukin-17. J. Biol. Chem..

[19] Langrish C.L., Chen Y., Blumenschein W.M., Mattson J., Basham B., Sedgwick J.D., McClanahan T., Kastelein R.A., Cua D.J. (2005). IL-23 drives a pathogenic T cell population that induces autoimmune inflammation. J. Exp. Med..

[20] Fouser L.A., Wright J.F., Dunussi-Joannopoulos K., Collins M. (2008). Th17 cytokines and their emerging roles in inflammation and autoimmunity. Immunol. Rev..

[21] Singh R.P., Hasan S., Sharma S., Nagra S., Yamaguchi D.T., Wong D.T., Hahn B.H., Hossain A. (2014). Th17 cells in inflammation and autoimmunity. Autoimmun. Rev..

[22] McGeachy M.J., Cua D.J., Gaffen S.L. (2019). The IL-17 Family of Cytokines in Health and Disease. Immunity.

[23] Neurath M.F., Finotto S. (2011). IL-6 signaling in autoimmunity, chronic inflammation and inflammation-associated cancer. Cytokine Growth Factor Rev..

[24] McGeachy M.J., Bak-Jensen K.S., Chen Y., Tato C.M., Blumenschein W., McClanahan T., Cua D.J. (2007). TGF-beta and IL-6 drive the production of IL-17 and IL-10 by T cells and restrain T(H)-17 cell-mediated pathology. Nat. Immunol..

[25] Yen D., Cheung J., Scheerens H., Poulet F., McClanahan T., McKenzie B., Kleinschek M.A., Owyang A., Mattson J., Blumenschein W. (2006). IL-23 is essential for T cell-mediated colitis and promotes inflammation via IL-17 and IL-6. J. Clin. Investig..

[26] Eugster H.P., Frei K., Kopf M., Lassmann H., Fontana A. (1998). IL-6-deficient mice resist myelin oligodendrocyte glycoprotein-induced autoimmune encephalomyelitis. Eur. J. Immunol..

[27] Okuda Y., Sakoda S., Bernard C.C., Fujimura H., Saeki Y., Kishimoto T., Yanagihara T. (1998). IL-6-deficient mice are resistant to the induction of experimental autoimmune encephalomyelitis provoked by myelin oligodendrocyte glycoprotein. Int. Immunol..

[28] Samoilova E.B., Horton J.L., Hilliard B., Liu T.S., Chen Y. (1998). IL-6-deficient mice are resistant to experimental autoimmune encephalomyelitis: Roles of IL-6 in the activation and differentiation of autoreactive T cells. J. Immunol..

[29] Khader S.A., Pearl J.E., Sakamoto K., Gilmartin L., Bell G.K., Jelley-Gibbs D.M., Ghilardi N., Desauvage F., Cooper A.M. (2005). IL-23 Compensates for the Absence of IL-12p70 and Is Essential for the IL-17 Response during Tuberculosis but Is Dispensable for Protection and Antigen-Specific IFN-{gamma} Responses if IL-12p70 Is Available. J. Immunol..

[30] Hölscher C., Atkinson R.A., Arendse B., Brown N., Myburgh E., Alber G., Brombacher F. (2001). A protective and agonistic function of IL-12p40 in mycobacterial infection. J. Immunol..

[31] Ladel C.H., Blum C., Dreher A., Reifenberg K., Kopf M., Kaufmann S.H. (1997). Lethal tuberculosis in interleukin-6-deficient mutant mice. Infect. Immun..

[32] Scheller J., Chalaris A., Schmidt-Arras D., Rose-John S. (2011). The pro- and anti-inflammatory properties of the cytokine interleukin-6. Biochim. Biophys. Acta.

[33] Betz U.A., Bloch W., van den Broek M., Yoshida K., Taga T., Kishimoto T., Addicks K., Rajewsky K., Muller W. (1998). Postnatally induced inactivation of gp130 in mice results in neurological, cardiac, hematopoietic, immunological, hepatic, and pulmonary defects. J. Exp. Med..

[34] Kopf M., Brombacher F., Kohler G., Kienzle G., Widmann K.H., Lefrang K., Humborg C., Ledermann B., Solbach W. (1996). IL-4-deficient Balb/c mice resist infection with Leishmania major. J. Exp. Med..

[35] Behrends J., Renauld J.-C., Ehlers S., Hölscher C. (2013). IL-22 Is Mainly Produced by IFNγ-Secreting Cells but Is Dispensable for Host Protection against Mycobacterium tuberculosis Infection. PLoS ONE.

[36] McLoughlin R.M., Jenkins B.J., Grail D., Williams A.S., Fielding C.A., Parker C.R., Ernst M., Topley N., Jones S.A. (2005). IL-6 trans-signaling via STAT3 directs T cell infiltration in acute inflammation. Proc. Natl. Acad. Sci. USA.

[37] Schreiber T., Ehlers S., Heitmann L., Rausch A., Mages J., Murray P.J., Lang R., Holscher C. (2009). Autocrine IL-10 induces hallmarks of alternative activation in macrophages and suppresses antituberculosis effector mechanisms without compromising T cell immunity. J. Immunol..

[38] Murray P.J., Wang L., Onufryk C., Tepper R.I., Young R.A. (1997). T cell-derived IL-10 antagonizes macrophage function in mycobacterial infection. J. Immunol..

[39] MacMicking J.D., Nathan C., Hom G., Chartrain N., Fletcher D.S., Trumbauer M., Stevens K., Xie Q.W., Sokol K., Hutchinson N. (1995). Altered responses to bacterial infection and endotoxic shock in mice lacking inducible nitric oxide synthase. Cell.

[40] MacMicking J.D., Taylor G.A., McKinney J.D. (2003). Immune control of tuberculosis by IFN-gamma-inducible LRG-47. Science.

[41] Korn T., Mitsdoerffer M., Croxford A.L., Awasthi A., Dardalhon V.A., Galileos G., Vollmar P., Stritesky G.L., Kaplan M.H., Waisman A. (2008). IL-6 controls Th17 immunity in vivo by inhibiting the conversion of conventional T cells into Foxp3^+^ regulatory T cells. Proc. Natl. Acad. Sci. USA.

[42] Yamashita T., Iwakura T., Matsui K., Kawaguchi H., Obana M., Hayama A., Maeda M., Izumi Y., Komuro I., Ohsugi Y. (2011). IL-6-mediated Th17 differentiation through RORγt is essential for the initiation of experimental autoimmune myocarditis. Cardiovasc. Res..

[43] Dileepan T., Linehan J.L., Moon J.J., Pepper M., Jenkins M.K., Cleary P.P. (2011). Robust antigen specific th17 T cell response to group A Streptococcus is dependent on IL-6 and intranasal route of infection. PLoS Pathog..

[44] Okamoto Yoshida Y., Umemura M., Yahagi A., O’Brien R.L., Ikuta K., Kishihara K., Hara H., Nakae S., Iwakura Y., Matsuzaki G. (2010). Essential role of IL-17A in the formation of a mycobacterial infection-induced granuloma in the lung. J. Immunol..

[45] Umemura M., Yahagi A., Hamada S., Begum M.D., Watanabe H., Kawakami K., Suda T., Sudo K., Nakae S., Iwakura Y. (2007). IL-17-mediated regulation of innate and acquired immune response against pulmonary Mycobacterium bovis bacille Calmette-Guerin infection. J. Immunol..

[46] Zhou L., Ivanov I.I., Spolski R., Min R., Shenderov K., Egawa T., Levy D.E., Leonard W.J., Littman D.R. (2007). IL-6 programs T(H)-17 cell differentiation by promoting sequential engagement of the IL-21 and IL-23 pathways. Nat. Immunol..

[47] Wei L., Laurence A., Elias K.M., O’Shea J.J. (2007). IL-21 is produced by Th17 cells and drives IL-17 production in a STAT3-dependent manner. J. Biol. Chem..

[48] Lei L., Zhong X.N., He Z.Y., Zhao C., Sun X.J. (2015). IL-21 induction of CD4^+^ T cell differentiation into Th17 cells contributes to bleomycin-induced fibrosis in mice. Cell Biol. Int..

[49] Korn T., Bettelli E., Gao W., Awasthi A., Jäger A., Strom T.B., Oukka M., Kuchroo V.K. (2007). IL-21 initiates an alternative pathway to induce proinflammatory T(H)17 cells. Nature.

[50] Shi Y., Chen Z., Zhao Z., Yu Y., Fan H., Xu X., Bu X., Gu J. (2019). IL-21 Induces an Imbalance of Th17/Treg Cells in Moderate-to-Severe Plaque Psoriasis Patients. Front. Immunol..

[51] Tan Y., Chen W., Liu C., Zheng X., Guo A., Long J. (2019). Effect of IL-21 on the Balance of Th17 Cells/Treg Cells in the Pathogenesis of Graves’ Disease. Endocr. Res..

[52] Cheekatla S.S., Tripathi D., Venkatasubramanian S., Paidipally P., Welch E., Tvinnereim A.R., Nurieva R., Vankayalapati R. (2017). IL-21 Receptor Signaling Is Essential for Optimal CD4^(+)^ T Cell Function and Control of Mycobacterium tuberculosis Infection in Mice. J. Immunol..

[53] Booty M.G., Barreira-Silva P., Carpenter S.M., Nunes-Alves C., Jacques M.K., Stowell B.L., Jayaraman P., Beamer G., Behar S.M. (2016). IL-21 signaling is essential for optimal host resistance against Mycobacterium tuberculosis infection. Sci. Rep..

[54] Monin L., Griffiths K.L., Slight S., Lin Y., Rangel-Moreno J., Khader S.A. (2015). Immune requirements for protective Th17 recall responses to Mycobacterium tuberculosis challenge. Mucosal Immunol..

[55] Cho M.L., Kang J.W., Moon Y.M., Nam H.J., Jhun J.Y., Heo S.B., Jin H.T., Min S.Y., Ju J.H., Park K.S. (2006). STAT3 and NF-kappaB signal pathway is required for IL-23-mediated IL-17 production in spontaneous arthritis animal model IL-1 receptor antagonist-deficient mice. J. Immunol..

[56] Saunders B.M., Frank A.A., Orme I.M., Cooper A.M. (2000). Interleukin-6 induces early gamma interferon production in the infected lung but is not required for generation of specific immunity to Mycobacterium tuberculosis infection. Infect. Immun..

[57] Sodenkamp J., Behrends J., Forster I., Muller W., Ehlers S., Holscher C. (2011). gp130 on macrophages/granulocytes modulates inflammation during experimental tuberculosis. Eur. J. Cell Biol..

[58] Sodenkamp J., Waetzig G.H., Scheller J., Seegert D., Grötzinger J., Rose-John S., Ehlers S., Hölscher C. (2012). Therapeutic targeting of interleukin-6 trans-signaling does not affect the outcome of experimental tuberculosis. Immunobiology.

[59] Fasnacht N., Greweling M.C., Bollati-Fogolín M., Schippers A., Müller W. (2009). T-cell-specific deletion of gp130 renders the highly susceptible IL-10-deficient mouse resistant to intestinal nematode infection. Eur. J. Immunol..

[60] Rose-John S., Winthrop K., Calabrese L. (2017). The role of IL-6 in host defence against infections: Immunobiology and clinical implications. Nat. Rev. Rheumatol..

[61] Martinez A.N., Mehra S., Kaushal D. (2013). Role of interleukin 6 in innate immunity to Mycobacterium tuberculosis infection. J. Infect. Dis..

[62] Taylor G.A., Jeffers M., Largaespada D.A., Jenkins N.A., Copeland N.G., Vande Woude G.F. (1996). Identification of a novel GTPase, the inducibly expressed GTPase, that accumulates in response to interferon gamma. J. Biol. Chem..

[63] Lowenstein C.J., Padalko E. (2004). iNOS (NOS2) at a glance. J. Cell Sci..

[64] Redford P.S., Murray P.J., O’Garra A. (2011). The role of IL-10 in immune regulation during M. tuberculosis infection. Mucosal Immunol..

[65] Rottenberg M.E., Carow B. (2014). SOCS3 and STAT3, major controllers of the outcome of infection with Mycobacterium tuberculosis. Semin. Immunol..

