# Supplementary material for: IL-6 Is Not Absolutely Essential for the Development of a TH17 Immune Response after an Aerosol Infection with Mycobacterium tuberculosis H37rv
Source: Cells. 2020 Dec 22;10(1):9. doi: 10.3390/cells10010009 (PMC7822128; doi:10.3390/cells10010009)
Supplement: Supplementary file 1 [file cells-10-00009-s001.pdf]

IL-6 is not absolutely essential for the development of a TH17 immune response after an aerosol infection with *Mycobacterium tuberculosis* H37rv

Supplementary materials

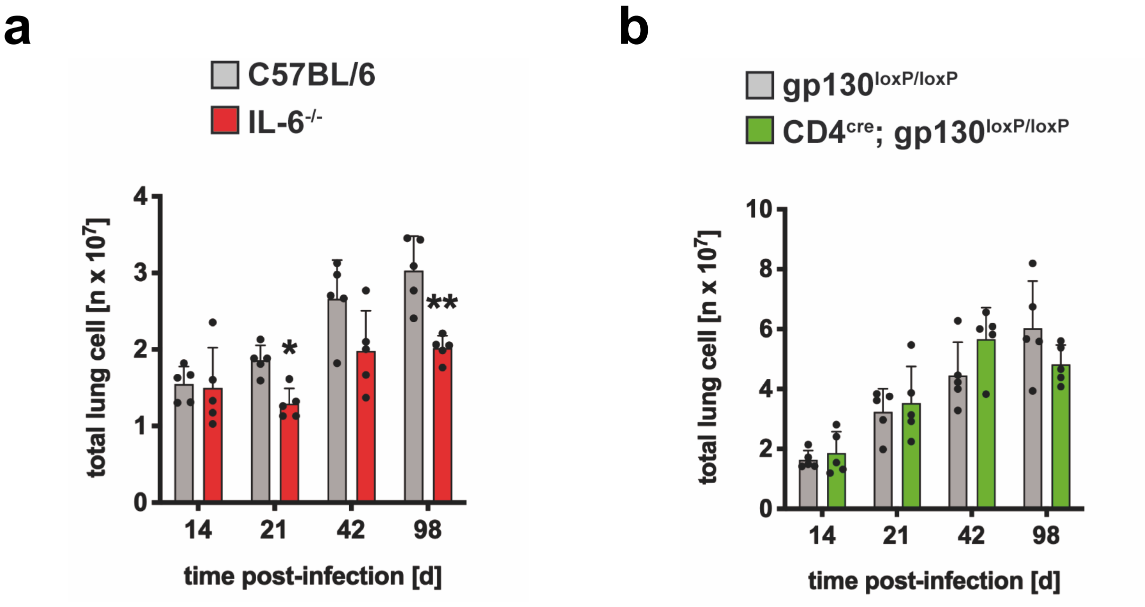

Figure S1. The number of lung cells during experimental TB. (a) C57BL/6 and IL-6$^{-/-}$ or **(b)** gp130$^{loxP/loxP}$ and CD4^{cre}; gp130$^{loxP/loxP}$ mice were infected with approximately 100 CFU Mtb via the aerosol route. At different time points lungs were removed, further processed and the number of living cells automatically counted. Data represent means and standard deviations of five mice. One experiment representative of two performed is shown. Statistical analysis was performed using the Mann-Whitney test defining differences between C57BL/6 and IL-6$^{-/-}$ mice as significant (*, p≤0.05; **, p≤0.01).

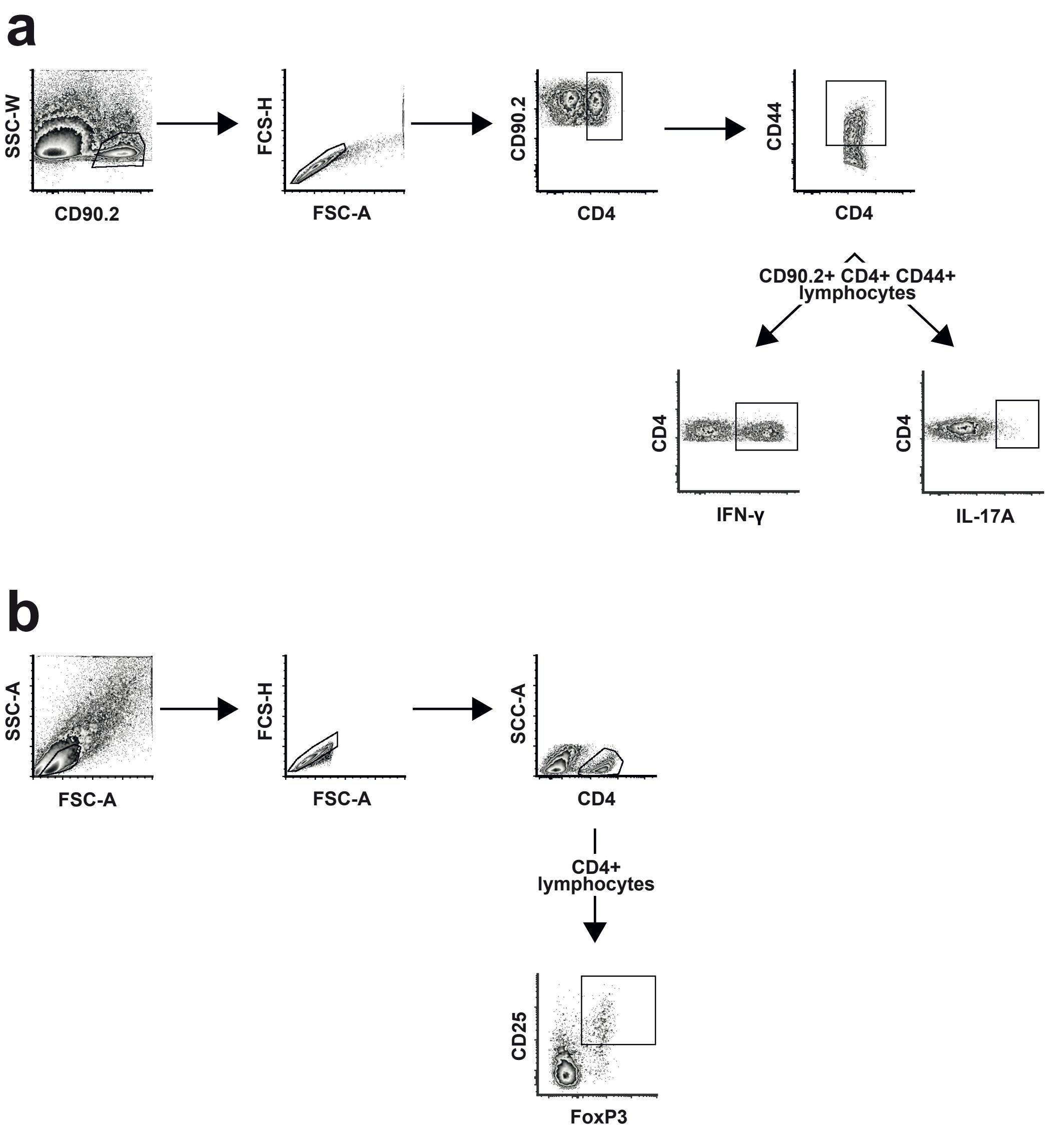

Figure S2. Gating strategies for intracellular detection of IFNγ, IL-17A and FoxP3⁺ by flow cytometry. Experimental mice were infected with approximately 100 CFU Mtb via the aerosol route. At different time points lungs were removed and further processed. **(a)** Gating strategy to evaluate frequencies of IFNγ- and IL-17A-producing cells out of $CD44^+$ $CD4^+$ $CD90.2^+$ cells. **(b)** Gating strategy to evaluate frequencies of $FoxP3^+CD25^+$ cells out of $CD4^+$ cells. Representative density plots analysed on day 42 are shown.

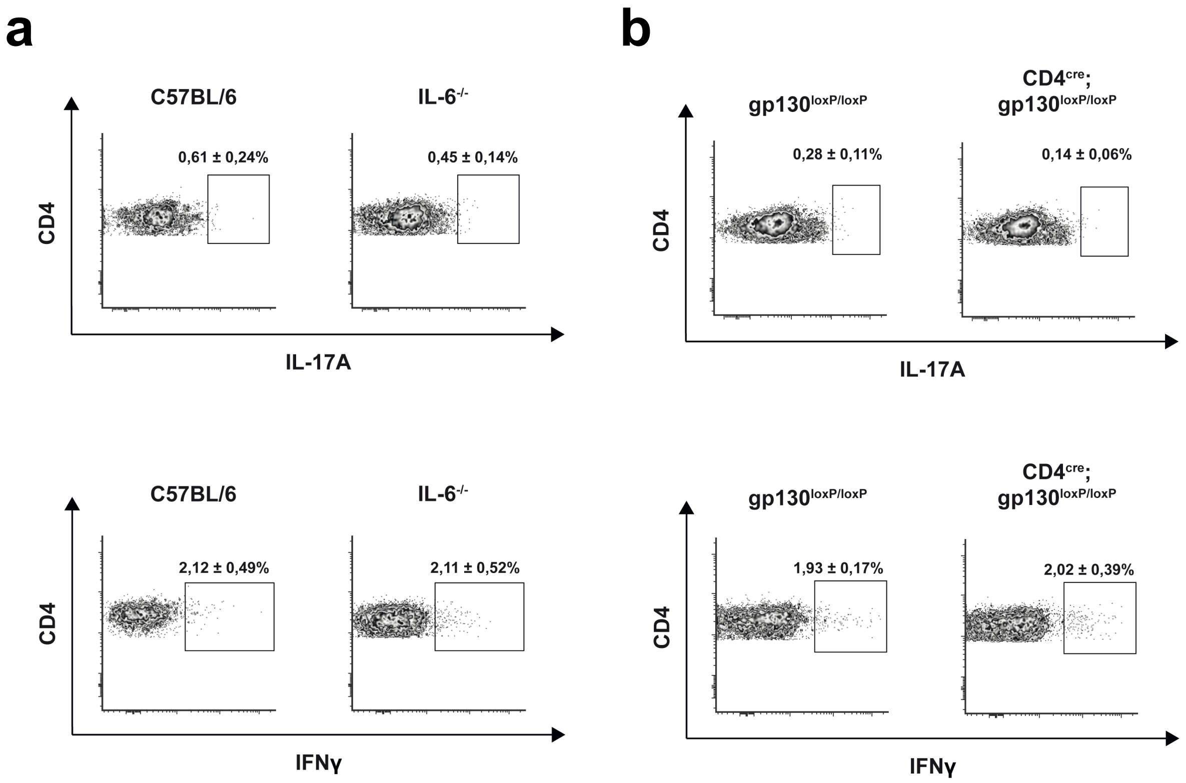

Figure S3. The TH1/TH17 immune response during experimental TB – gating of unstimulated cells. (a) C57BL/6 and IL-$6^{-/-}$ or **(b)** $gp130^{loxP/loxP}$ and $CD4^{cre}$; $gp130^{loxP/loxP}$ mice were infected with approximately 100 CFU Mtb via the aerosol route. At different time points, lungs were removed and further processed. The relative amount of IL-17A- and IFNγ-producing $CD4^+CD44^+CD90.2^+$ T cells was determined in single cell suspensions of lungs by flow cytometry without restimulation. Representative density plots of IL-17A-/IFNγ-producing $CD4^+CD44^+CD90.2^+$ T cells on day 42 are shown. Data represent means and standard deviations of five mice.

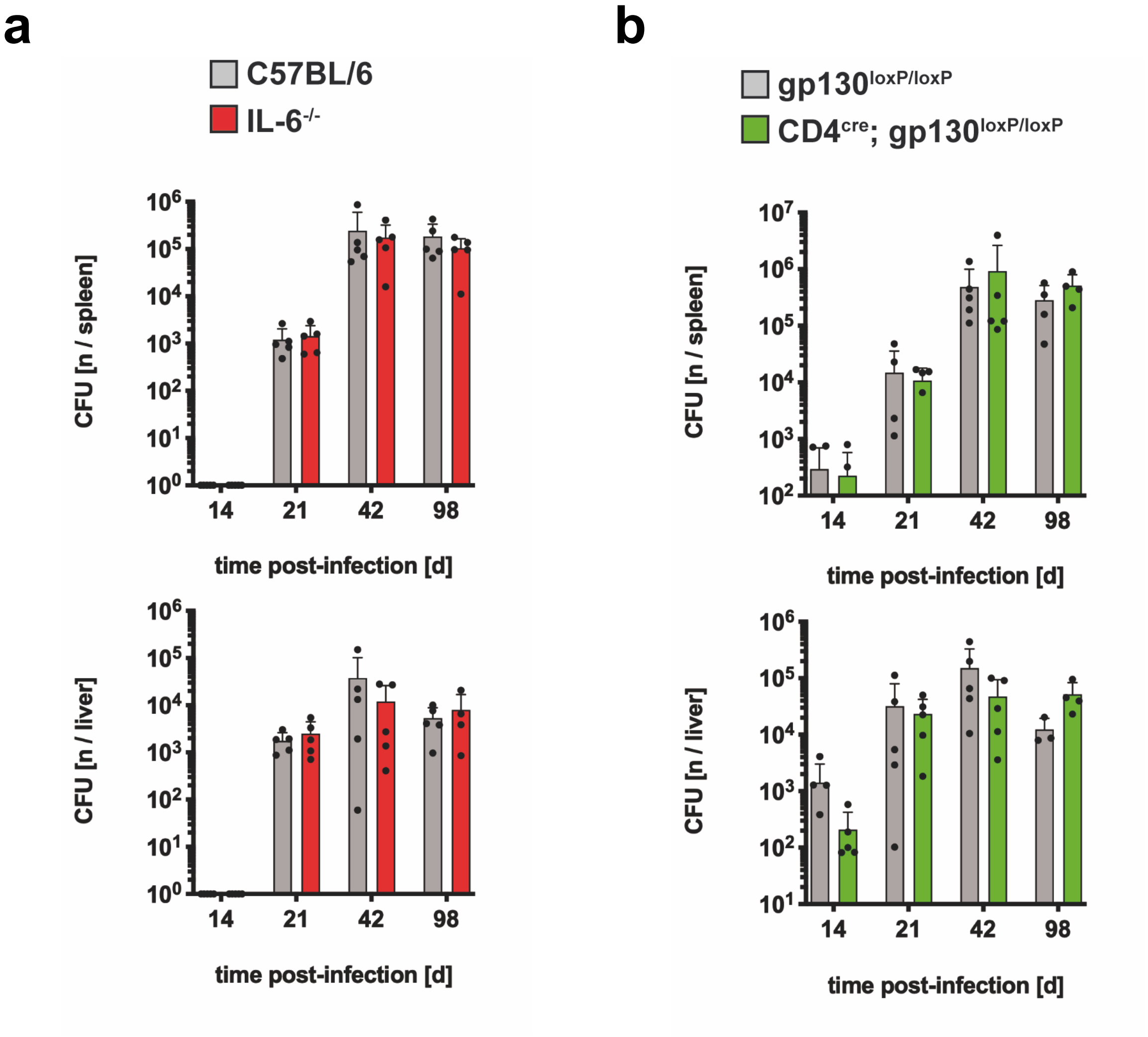

Figure S4. Bacterial loads in spleen and liver after infection with Mtb. (a) C57BL/6 and IL-6$^{-/-}$ or **(b)** gp130$^{loxP/loxP}$ and CD4^{cre}; gp130$^{loxP/loxP}$ mice were infected with approximately 100 CFU Mtb via the aerosol route. At the indicated time points, bacterial loads in the spleen and liver were determined. Data represent means and standard deviations of five mice per group. One experiment representative of two performed is shown.

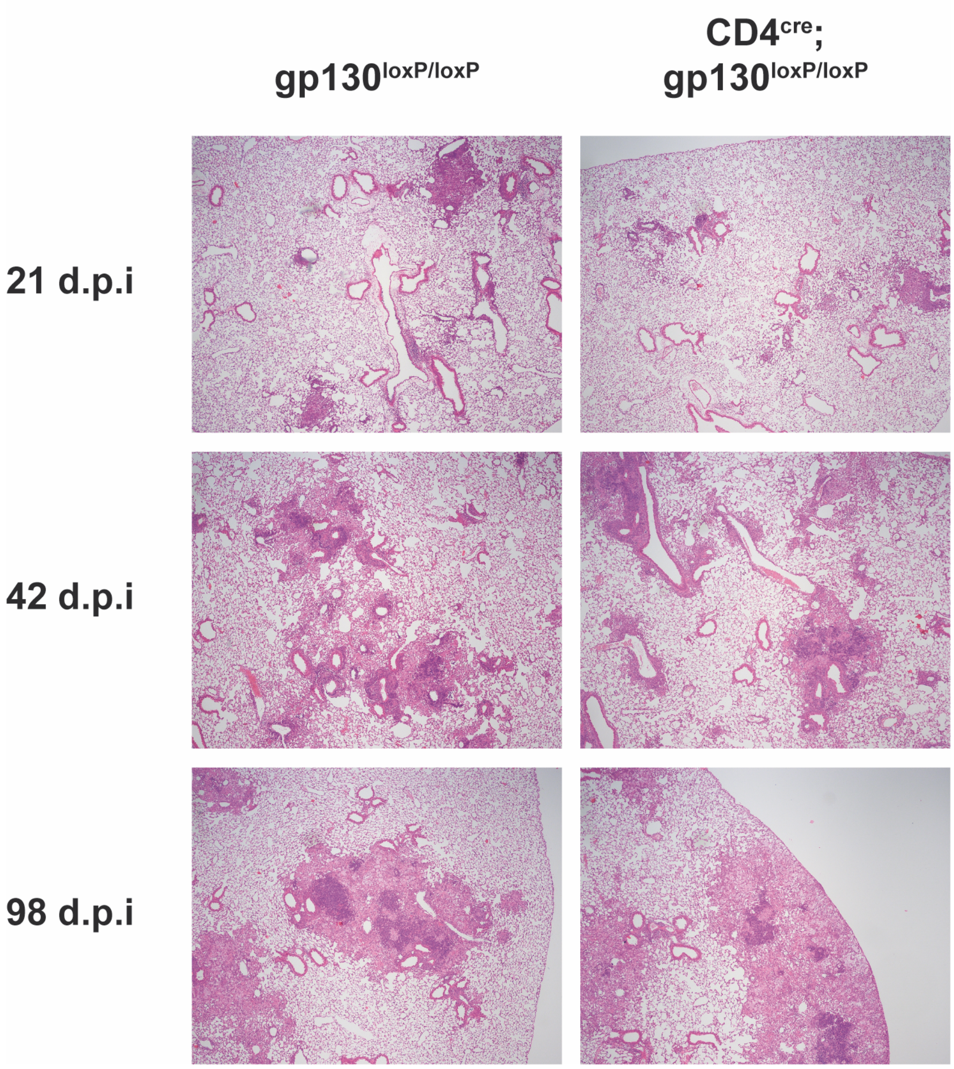

Figure S5. Histopathological changes in lungs of gp130$^{loxP/loxP}$ and CD4^{cre}; gp130$^{loxP/loxP}$ mice after infection with Mtb. gp130$^{loxP/loxP}$ and CD4^{cre}; gp130$^{loxP/loxP}$ mice infected with approximately 100 CFU Mtb via the aerosol route. At the indicated time points, lungs were fixed in formalin, embedded in paraffin, sectioned on a microtome and stained with hematoxilin/eosin. Representative photomicrographs of 5 mice per group are shown.
